# Mismatch Repair: From Preserving Genome Stability to Enabling Mutation Studies in Real-Time Single Cells

**DOI:** 10.3390/cells10061535

**Published:** 2021-06-18

**Authors:** Marina Elez

**Affiliations:** 1 Micalis Institute, INRAE, AgroParisTech, Université Paris-Saclay, 78350 Jouy-en-Josas, France; marina.elez@inrae.fr; 2 Laboratoire Jean Perrin (LJP), Institut de Biologie Paris-Seine (IBPS), CNRS, Sorbonne Université, F-75005 Paris, France

**Keywords:** mismatch repair, DNA replication errors, recombination, spontaneous mutations, fluorescence microscopy

## Abstract

Mismatch Repair (MMR) is an important and conserved keeper of the maintenance of genetic information. Miroslav Radman’s contributions to the field of MMR are multiple and tremendous. One of the most notable was to provide, along with Bob Wagner and Matthew Meselson, the first direct evidence for the existence of the methyl-directed MMR. The purpose of this review is to outline several aspects and biological implications of MMR that his work has helped unveil, including the role of MMR during replication and recombination editing, and the current understanding of its mechanism. The review also summarizes recent discoveries related to the visualization of MMR components and discusses how it has helped shape our understanding of the coupling of mismatch recognition to replication. Finally, the author explains how visualization of MMR components has paved the way to the study of spontaneous mutations in living cells in real time.

## 1. Introduction

The MMR system is an important DNA-repair process found in all three domains of life. It is conserved in almost all organisms, except for most Actinobacteria and Mollicutes, and parts of the archaea [1,2]. MMR involves several steps and a dozen proteins working in concert and surveys two critical aspects of DNA metabolism: DNA replication and recombination. MMR is an important contributor to the stability of genomes. Loss of its activity results in up to a thousand-fold increase in spontaneous point mutation rate, an increase in recombination, and high instability of short repeated sequences in organisms ranging from bacteria to humans. The observation of the latter phenotype [3,4] in some tumors led to the discovery that mutations in MMR genes cause hereditary nonpolyposis colorectal cancer, i.e., Lynch syndrome [5,6,7,8,9] and a significant fraction of sporadic cancers [10]. MMR proteins also act on several other cellular processes. MMR triggers apoptosis to DNA damage and can be hijacked by some cells for its mutagenic activity, for instance, during antibody diversification. Moreover, homologs of MMR proteins contribute to the accurate segregation of chromosomes during meiosis. These alternative roles will not be discussed further here, as they are reviewed in recent works [11,12,13,14]. In addition, some bacterial lineages have lost MMR genes, an observation that prompted the proposal that the loss of these genes may be advantageous. Indeed, MMR deficient mutants were shown to evolve readily in laboratory populations undergoing adaptation to a new environment [15,16]. The evolutionary implications of MMR genes are not considered here, either, and are reviewed elsewhere [17,18].

## 2. MMR Maintains Genome Stability by Editing DNA Replication and Recombination

### 2.1. Replication Editing

During DNA replication, MMR detects errors of nucleotide incorporation in the DNA molecule (mismatches, small insertion, and deletion loops) and recruits enzymes to excise and resynthesize the portion of the newly synthesized strand containing the error. The latter is a crucial feature of the MMR pathway [19,20]: if it were not able to discriminate the new strand from the old, it would correct only 50% of the errors, and the rest would be converted into mutations. How MMR differentiates between the two strands was one of the first questions that came to mind when the existence of such a DNA editing system was hypothesized in 1976 [21]. The mechanism was established quickly, forty years ago, for *Escherichia coli* [22,23]. This species encodes a Dam methyltransferase that catalyzes the post-replicative methylation of A at N6 position in d(GATC) palindromic sequences [24,25]. Because Dam methylation lags behind the replicative DNA polymerase, on the order of minutes, newly synthesized d(GATC)s are transiently unmethylated [26,27,28]. MMR exploits the transient hypomethylated state of the newly synthesized strand. It recognizes and cleaves the hemimethylated d(GATC) on the new unmethylated strand, whereas fully methylated d(GATC)s are fully resistant to cleavage [23,29,30,31]. Methyl-directed MMR (me-MMR) is restricted to a set of *E. coli* related gammaproteobacteria. It most probably evolved from a MMR pathway that does not use DNA methylation, functioning in most other organisms [2]. How DNA strand discrimination works in canonical MMR remains an open question. Recent evidence suggests that pre-existing nicks in the newly synthesized strand, natural intermediates during replication, may serve as a signal [32].

**Figure 1:**
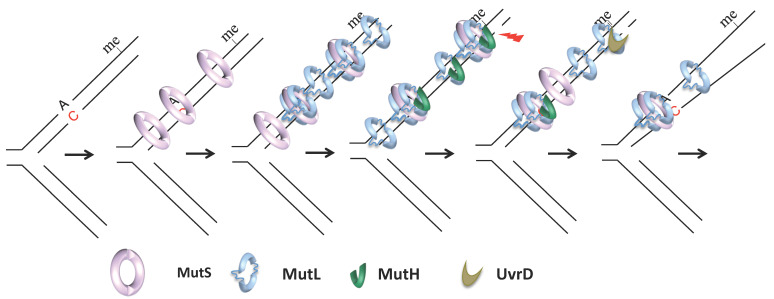
MMR mechanism of *E. coli*. Replication error repair in *E. coli* is presumed to be carried out by the following sequence of steps: MutS dimer binds to a mismatch; stabilization of the ATP-bound MutS state; MutS slides away from the mismatch; additional copies of MutS load onto the mismatch -> one or several MutL dimers bind to sliding MutS; MutS or MutL can dissociate from diffusing MutS–MutL complexes -> MutH binds to diffusing MutL or MutS–MutL complexes; physical interaction between MutH and MutL activates MutH endonuclease activity; activated MutH incises the newly synthesized strand at the unmethylated d(GATC) sequence -> MutL and UvrD assemble near the single-strand break -> binding of UvrD to MutL allows unwinding of the portion of the newly synthesized strand beyond the mismatch -> the unwound portion of the new strand is resected; DNA polymerase resynthesizes the excised strand, and DNA ligase seals the nick (not shown); me, methylated GATC sequence (old strand).

MutS, MutL, MutH, and UvrD, the critical players of *E. coli* MMR, were identified in 1980 by Radman and Glickman [33,34,35] (Figure 1). Previous studies had established that inactivation of some of these genes resulted in the increased mutation rate in bacteria [36,37,38]. Radman and Glickman exploited MMR non-directedness in Dam-deficient cells (i.e., in the absence of methylation, MMR cleaves the template and the new strand) to design a clever screen. They knew that *dam* cells die in the presence of 2-aminopurine (2-AP), which increases the load of mismatches in cells, because MMR non-directedness in this condition results in multiple double-strand breaks that overload the cell’s repair capacity. Radman and Glickman reasoned that inactivation of MMR should restore the viability of *dam* cells in the presence of 2-AP. Screening for this viability allowed them to identify key MMR genes. The availability of MMR inactivation mutants enabled the purification and biochemistry of individual components in Paul Modrich’s laboratory, leading to the elucidation of each protein’s role and paved a way for Paul Modrich to win the Nobel prize in Chemistry in 2015 for his work on mechanistic studies of MMR.

MutS is a homodimeric ABC family ATPase that recognizes and binds to single-base mismatches and insertions, deletions up to four nucleotides [39,40,41,42] (Figure 1). The efficiency with which MutS binds to different base–base and indel mismatches varies by more than ten-fold in vitro [43]. In vivo, the most efficiently repaired mismatches correspond to the most frequent replication errors (transitions and frameshifts) [44,45,46]. In solution, MutS exists in a balance of dimers and tetramers, but the importance of tetramers in MMR is currently unclear [47,48]. Like MutS, MutL is a weak homodimeric GHKL family ATPase [49,50]. It binds to MutS, which requires mismatch and ATP [51,52,53] and to downstream components MutH and UvrD, which allows their activation and the initiation of downstream steps [54] (Figure 1). Its main function seems to be the coordination of the different steps of MMR, hence its name of matchmaker protein. MutH is a monomeric endonuclease that evolved from the type II endonuclease [55]. It makes single-stranded nicks 5′ of G in unmethylated d(GATC) sequences that can be up to a 1 to 2 kilobase away, and either 5′ or 3′ of a mismatch [30,31,56,57,58,59]. The origin distal d(GATC) site between the mismatch and the replication fork is used in vivo, suggesting that bidirectionality at the level of strand excision transduces into unidirectionality with respect to chromosome replication [60]. Activation of MutH endonuclease activity requires physical interaction with MutL in a mismatch, MutS, and ATP-dependent manner [49,56,61]. Nicks made by MutH serve as an entry point for 3′–5′ helicase UvrD, which, if stimulated by MutL [62,63,64,65] unwinds the portion of the nicked strand from the nick toward the mismatch (Figure 1). UvrD loads onto the template or the newly synthesized strand depending on the orientation of the nicked d(GATC) relative to the mismatch. One or more exonucleases then resect the unwound strand: Exo I, VII, or X may act in the 3′–5′ excision, while RecJ or ExoVII may act in the 5′–3′ excision [66,67]. In vitro reconstruction of methyl-directed MMR showed that DNA polymerase III could resynthesize the excised strand and DNA-ligase seal the remaining nick [68].

Work in eukaryotic models (*Saccharomyces cerevisiae*, *Xenopus laevis*, and human cells) revealed genes encoding eukaryotic MMR proteins and led to the in vitro reconstitution of human MMR in the mid-2000s [69,70]. The eukaryotic MMR has several key differences from its prokaryotic counterpart [71]. First, eukaryotes form several heterodimers of MutS and MutL homologs, which exhibit some functional specialization. The MutS homolog MutSalpha (MSH2–MSH6 heterodimer) is specialized for mismatches and 1 or 2 base-pair loops. MutSbeta (MSH2–MSH3 heterodimer) has a minor role as it only repairs some larger insertion and deletion loops. The MSH4–MSH5 heterodimer does not participate in replication editing. Instead, it is required for heteroduplex stabilization during meiotic recombination [11,72]. Eukaryotic homologs of MutL include: MutLalpha (MLH1–PMS1 or MLH1–PMS2 heterodimer in yeast and human cells, respectively), which plays a major role and works with MutSalpha; MutLbeta (MLH1–MLH2 or MLH1–PMS1 heterodimer, in yeast and human cells, respectively), which is considered an accessory factor of MutLalpha; and MutLgamma (MLH1–MLH3 heterodimer), which works with MutSbeta and with MSH4–MSH5 in the processing of recombination intermediates. Second, eukaryotes do not have a MutH homolog. Instead, human PMS2, its yeast equivalent PMS1, and MLH3 possess a weak endonuclease activity [32,73]. Furthermore, eukaryotic MMR does not require helicases and relies on Exonuclease I or replicative polymerase activity for strand excision and displacement.

### 2.2. Recombination Editing

The idea that a dedicated repair system could repair DNA replication errors emerged after Robin Holliday hypothesized in 1964 the existence of a cellular system dedicated to the repair of mismatches arising from recombination during meiosis. Indeed, meiotic recombination between two non-identical, parental DNA molecules leads to DNA heteroduplexes with mismatches. Holliday hypothesized that the MMR-like repair process could repair these mismatches, which provided an explanation for the non-Mendelian inheritance of alleles. In addition to meiosis, somatic recombination processes can lead to the formation of DNA heteroduplexes containing mismatches. For example, double-strand breaks (due to collapsed/stalled replication forks or simultaneous excision DNA repair events on opposite DNA strands) can be repaired by recombination involving repetitive, non-identical DNA sequences. In addition, DNA transferred between cells by conjugation, transduction, or transformation can integrate into the host genome through recombination. If the transferred and host DNA are not 100% identical at the sequence level, this can also result in DNA heteroduplexes with mismatches. The outcome of the MMR activity can vary depending on the recombination pathway used (e.g., synthesis-dependent strand annealing, break-induced replication, or single-strand annealing), the number of mismatches, or the reaction step. MMR can repair mismatches, and, as Holliday suggested, this can result in gene conversion: in meiosis, the genetic information of one genotype replaces an allelic phenotype [74].

Alternatively, MMR-mediated detection of mismatches in DNA heteroduplex can cause abortion of recombination by rejection of the DNA heteroduplex. Radman and colleagues demonstrated this in 1989 [75] by studying conjugation between *E. coli* and *Salmonella typhimurium*, which are 85% identical at the DNA sequence level. They found that, in the absence of MMR, the frequency of homologous recombination during conjugation between *E. coli* and *S. typhimurium* increases 1000-fold [76,77]. These results suggested that MMR introduces a barrier to recombination between species. Indeed, subsequent studies revealed that transduction and transformation are also negatively regulated by MMR [78,79,80]. In addition, MMR also negatively regulates recombination events involving non-identical repetitive sequences in genomes that can cause deletions, inversion, and other types of chromosomal rearrangements [81,82,83,84]. Based on these findings, new roles for MMR have been proposed: MMR acts as a barrier to chromosomal rearrangements and between species [85,86,87,88].

MMR repair on recombination intermediates is similar to replication error repair (except perhaps for the coupling to the replication machinery). In contrast, how MMR processing of mismatches in recombination intermediates leads to suppression of recombination is less well understood. The latter has been most extensively studied for a strand-exchange-initiated homologous recombination process, catalyzed by a nucleoprotein filament consisting of a single-stranded DNA, the strand exchange protein RecA (or its eukaryotic homolog Rad51), and ATP. The strand exchange results in a joint heteroduplex DNA molecule. In the following step, the heteroduplex expands by branch migration. In vivo experiments from Radman’s laboratory established that in *E. coli*, MMR mediated suppression of homologous recombination, in contrast to the editing of replication errors, is unequally dependent on four key MMR proteins [75,89]. Inactivation of *mutS* and *mutL* results in a very large and similar increase in recombination level. The inactivation of either *mutH* or *uvrD*, acting downstream, results in a lower stimulation of recombination, while inactivation of both genes produces an effect similar to the inactivation of *mutS* or *mutL*. The latter suggested that MutH and UvrD may act at different times during anti-recombination, with UvrD probably at earlier steps, and MutH at a later stage when de novo DNA synthesis is initiated. In yeast, Sgs1 and Srs2 helicases that do not play a role in replication editing participate in MMR-mediated suppression of recombination [90,91,92]. However, it is not known which helicases participate in this process in higher eukaryotes. In vitro studies using *E. coli* proteins suggest that MutS or MutL do not affect strand exchange initiation but act at the later branch migration step by inhibiting heteroduplex extension [93,94]. Work with human MSH2–MSH6 determined that it efficiently recognizes mismatches in recombination intermediates [95]. MutS alone is sufficient to block heteroduplex extension, but MutL enhances inhibition [93] even in the presence of the branch migration stimulator RuvAB [96]. A similar role for MutL is also suggested by an in vivo study of a heteroduplex size in *E. coli* as a function of the cellular MutL level [82]. Stalled strand exchange intermediates are potentially toxic and could be removed by the UvrD helicase [97].

## 3. MMR Mechanism

Biochemistry, crystallography, and biophysics studies of individual MMR proteins and their interactions have significantly advanced our understanding of the MMR mechanism. Some of the most pressing questions are how MutS scans the DNA in search of mismatches and how MutS binding to a mismatch leads to excision at the distal strand discrimination site. Models proposed over the past decades to explain the action at a distance during MMR have been hotly debated. The characterized mechanism of action of MutS provides a basis for models, as mechanistic analysis of MutL is only in its early stages.

The structures of MutS and its homologs revealed that these dimers form a ring around the DNA: two monomers connect at the bottom and top of the dimer and are separated in the middle by two large holes that can accommodate double-stranded DNA [41,42,98,99]. Each monomer contains seven distinct domains, of which only two are functional: DNA binding and ATPase domains located at the top and bottom of the monomer, respectively [100]. Each monomer contributes differentially to DNA binding, and two monomers have an alternating cycle of ATP binding and hydrolysis [101]. The MutS domains reorganize after DNA binding or ATP binding and hydrolysis, allowing for different conformational states to be generated and switched between [102,103]. Each state presumably has a necessary property that enables catalysis of a particular repair step, initially mismatch recognition and later coordination between mismatch recognition and the initiation of downstream strand excision via recruitment of MutL. In solution, for instance, ATP cycling-induced reorganization of the DNA binding domains leads to dynamic open and closed states of MutS. The open state allows MutS to load onto DNA, while MutS binding to DNA stabilizes the closed state. Binding to a mismatch provokes the stabilization of ATP-bound MutS state that can slide on DNA, thus known as the sliding clamp state [104,105,106].

MutL dimers also form a ring around the DNA. The crystal structure of intact MutL or its homologs has not been resolved. However, C- and N-terminal domains of MutL and its homologs have been crystallized separately [49,50,107,108,109,110]. The C-terminal domain is a primary dimerization interface. It contains, in organisms lacking a MutH homolog, an endonuclease activity. The N-terminal domain, connected to the C-terminal by a flexible linker, contacts MutS and DNA and contains a weak ATPase activity that is stimulated by MutL binding to DNA [49]. ATP binding induces dimerization of the N-terminal domain and leads to the formation of a central channel that can encircle double-stranded DNA. ATP binding significantly increases MutH endonuclease activity and UvrD helicase processivity. MutL likely has a complex ATPase cycle as MutS, and MutL might also adopt different conformation states upon ATP binding and hydrolysis [111].

Recent in vitro single-molecule tracking and single-molecule FRET studies of MutS and its homologs were instrumental to our current understanding of the MutS search mechanism: how MutS scans DNA before mismatch recognition [112,113,114,115,116]. These studies revealed that MutS binds to DNA via 3D diffusion and moves afterward along the DNA helix pitch via 1D diffusion associated with rotation. During the search for mismatches, MutS establishes non-specific contacts with DNA. Atomic force microscopy suggested that this process allows probing the bendability of DNA. The distortion introduced by a mismatched base pair results in an increased DNA flexibility [117]. Scanning for increased DNA flexibility might allow MutS to discriminate between perfectly matched DNA and DNA with a mismatch [117,118,119,120]. Crystallographic studies provided the molecular details of mismatch recognition and revealed that only two conserved residues of one monomer participate in this interaction [121]. MutS might sample the increased DNA flexibility by inserting a conserved phenylalanine at each base pair, and conserved glutamate could play a role in stabilizing the bend. DNA kinking upon mismatch recognition could provoke DNA push-back to release induced DNA stress and result in DNA unbending and the formation of a mobile MutS clamp that can slide away [122]. In this state, MutS is in non-continuous contact with the DNA and moves along the DNA with 1D diffusion that does not include a rotational component [112,114,115,116]. The latter enables for faster movement of MutS compared to the search MutS state.

At least three models have been proposed to account for the subsequent MMR step that depends on MutL and results in the establishment of a communication between a mismatch and a distant strand discrimination site. Unlike MutS, MutL yeast homolog MLH1-PMS1 can hop between different DNA sites through transient and rapid dissociation and re-association with the DNA [114,123]. This property might help its search for MutS upon MutS binding to a mismatch. MutL binding to MutS could stabilize MutS on a mismatch after MutS binding to ATP but before its conversion to a sliding clamp state [53,105,124]. This finding is compatible with a recent single-molecule FRET study of *Thermus aquaticus* MutS and MutL [125]. MutL mediated stabilization of mismatch bound MutS is central to the stationary or static transactivation model, which postulates that interaction with the strand discrimination signal occurs due to space collisions [41,49,105,126,127]. However, another single-particle tracking study, which used *E. coli* MMR proteins, revealed that MutL associates with MutS after its release from the mismatch [128]. The association between MutL and MutS was random along the DNA (Figure 1). The MutS–MutL complex diffuses with a 1D rotational mechanism, similar to the diffusion of MutS during the search phase, but with a ten-fold lower rate and oscillates between association and dissociation states. Alternatively, one of the proteins dissociates, leaving diffusing MutS and/or MutL clamps on DNA (Figure 1). That MutS and its homologs can slide away upon binding to a mismatch was observed for the first time more than 20 years ago and gave rise to the translocation models for MMR [52,129,130]. The active translocation model proposes that the MutS–MutL complex, or MutS alone, moves along DNA using free energy released from ATP hydrolysis. Such ATP hydrolysis-dependent movement can result in loop formation that might bring the mismatch close to the strand discrimination signal [52,131]. The alternative molecular switch model, which better fits recent *E. coli* single-molecule data, postulates that MutS conformational changes induced by ATP binding allow the protein to slide along the DNA passively [104,130,132]. Both active translocation and molecular switch models are consistent with the observation that a protein roadblock placed on DNA between a mismatch and an incision site can inhibit the repair substantially [133,134,135]. Finally, remote signaling may not be accomplished by a single mechanism, and both the stationary and mobile models might have their own advantages. The first could allow the localization of the cut in the mismatch vicinity, limiting excessive excision and re-synthesis. On the other hand, mobile proteins could release the mismatch, allowing for the loading of additional MutS and MutL proteins (Figure 1). Multiple MutS and MutL loading creates redundancy in the process allowing overcoming the non-productivity of MMR complexes that dissociate from DNA or cannot find the strand incision signal [136,137] (Figure 1).

Finally, recent single-molecule studies shed new light on the later steps of MMR, subject to less intensive investigation than MMR initiation [128,138]. These studies have shown that sliding MutL or MutL–MutS can recruit MutH, and a new complex continues to diffuse albeit at a lower rate (Figure 1). On the other hand, MutL and UvrD interaction is not random along DNA. These proteins assemble near single-strand breaks (Figure 1). MutL binding to UvrD significantly increases the UvrD unwinding processivity and DNA unwinding. Furthermore, DNA unwinding is rarely followed by exonuclease digestion. This finding is consistent with previous genetic studies showing that cells deficient for four *E. coli* single-stranded DNA exonucleases involved in MMR show only a modest mutation rate increase [67]. The exonucleases could be unnecessary because multiple MutL–MutH complexes could allow multiple incision events. This proposal is compatible with an idea first introduced by Wanger and Radman in 1986 that multiple-strand incision events on adjacent d(GATC) sites could occur during a single repair reaction [19].

## 4. Visualization of MMR Proteins in Live Cells

MMR protein localization studies in live cells initiated in the 2000s have completed our understanding of the MMR mechanism in vivo. In particular, they shaped our knowledge on the coupling of mismatch recognition to replication. They also challenged the idea of equal stoichiometry of MutS and MutL during MMR, assumed by all MMR models mentioned above. The fact that more MutL than MutS might be involved in each MMR reaction was first suggested by Paul Modrich to explain the observation of a dramatic increase in DNA footprint following MutL addition to a reaction containing MutS and heteroduplex DNA [20,51,105]. Moreover, the study of the dependence of the mismatch–MutS–MutL complex formation on the DNA length led to the proposal that it might involve the polymerization of MutL along DNA [139]. Subsequently, in 2007, Radman and colleagues raised the possibility of more MutL than MutS per single mismatch [140] to account for the phenomenon of MMR saturation in *E. coli*, reported in the 1980s by Radman’s laboratory and afterward by several other groups [141,142,143,144,145]. MMR system has a limited capacity to repair DNA mismatches: it saturates when the number of mismatches increases in the cell above a certain threshold due to MutL but not (or exceptionally) to MutS titration [146]. The engagement of more MutL than MutS proteins on the DNA mismatches was suggested after examining and rejecting several alternative explanations for MutL limitation. For instance, both MutL and MutS are present in cells at equimolar concentrations [147]. Furthermore, the conditions leading to MutL limitation do not cause MutL inactivation by degradation of MutL protein (Elez, M. and Radman, M., unpublished data).

The first study reporting MMR protein localization in lived cells was performed in *Bacillus subtilis* [148]. It involved fluorescently tagged MutS–GFP (or –YFP) that retained most wild-type MutS activity and an almost non-functional MutL–GFP. In *B. subtilis*, MutS–GFP is associated with the chromosome in all cells, while MutS–GFP and MutL–GFP form discrete foci in a small subset of cells. Besides *B. subtilis*, MMR complexes were also visualized in two other unicellular organisms, *E. coli* and *S. cerevisiae*, using fully functional MutS and MutL (for *E. coli*) or MSH6, PMS1, and MLH2 (for *S. cerevisiae*) fused to different fluorescent proteins (EGFP, YFP, CFP, mCherry, mYPET, 4GFP, TdTomato, and GFP) [140,149,150,151,152,153,154]. In *S. cerevisiae*, MMR fluorescent fusions were expressed at native levels [150,151] whereas different *E. coli* works examined either overexpression from a plasmid [149] or a unique chromosomal location [140,152] or expression at the native level [153,154]. These studies confirmed that both MutS and MutL and their homologs explored in *S. cerevisiae* localize into foci, and these and subsequent studies have focused on their characterization [155].

MutS foci formation was intensively investigated in *B. subtilis* and *S. cerevisiae*. The conventional epifluorescence microscopy-based study found that 5% of *B. subtilis* wild-type cells had a MutS focus. The percentage of cells with MutS foci increased to 43% after 2-AP treatment, and half of these co-localized with DNA polymerase foci [148]. *B. subtilis* MutS location and dynamics in live cells were further investigated by single-molecule super-resolution imaging [156]. MutS molecules with different diffusion rates were observed: half of the molecules passing by the replisome slowed down and dwelled there for at least 188 ms. The other half diffused freely within the cell. These results suggested that MutS enrichment around the replisome is much higher than established by conventional methods. This enrichment occurs independently of mismatch recognition and depends in part on interaction with the beta sliding clamp, the processivity factor of DNA polymerase [156,157,158]. This proximity to the replisome is critical for MutS to locate mismatches: it could improve mismatch recognition over the 3D + 1D diffusion-based search mechanisms. After mismatch detection, the diffusion rate of MutS increases: it remains associated with a mismatch and is thus carried away from the replisome until MutL is recruited [156,159]. Another argument for an intimate relationship between the replicating DNA polymerase complex and MMR in vivo is a demonstration by Klocko et al. of a MutS dependent decrease in the fraction of cells with foci of the primer maturation polymerase DnaE [160]. Similar to *B. subtilis*, investigation of MSH6 foci in *S. cerevisiae* suggested that MSH2–MSH6 foci are present at replication lforks, as evidenced by co-localization between MSH6–mCherry and different replication fork components (Pol2, Pol30, Pol1, and Pol3) [150]. Moreover, *S. cerevisiae* MSH6 foci formation requires interaction with the eukaryotic sliding clamp PCNA [150]. Finally, the abundance of *S. cerevisiae* MSH6 foci was unchanged in the condition where the number of mismatches increased in the cell, indicating that MSH2–MSH6 are present at replication sites at constitutive levels independent of mispaired bases [150].

**Figure 2:**
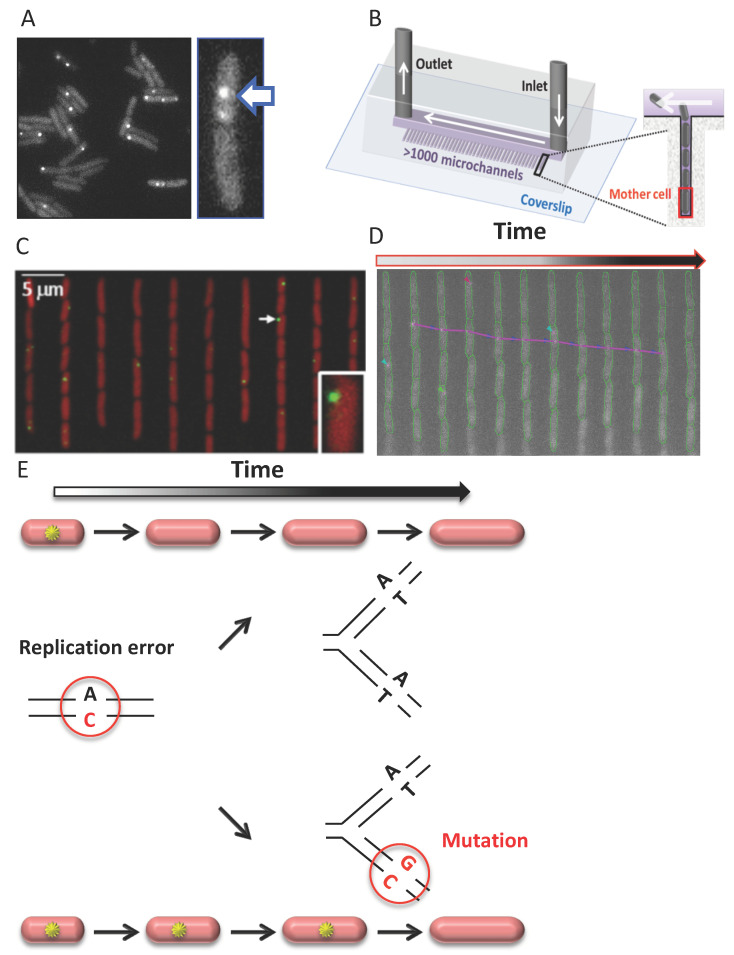
Visualization of MutL in single cells allows detection of replication errors and nascent mutations. (**A**) MMR MutL protein tagged with a yellow fluorescent protein (YFP) accumulates on DNA around replication errors, creating a fluorescent focus (arrow). (**B**) The microfluidic device *mother machine* enables tracking the accumulation of fluorescent foci of MutL in single cells under controlled conditions over more than 100 cell divisions. (**C**) Representative image of a *mother machine* experiment showing growing *E. coli* cells expressing YFP–MutL and tdCherry. Overlay of red and yellow fluorescence images is shown. Inset: magnified image of a cell with a YFP–MutL focus. (**D**) Kymograph of a single microchannel over time, imaged in yellow fluorescence. The time interval between two successive images is 2 min. YFP–MutL foci are segmented (magenta contours) and tracked (colored arrows). Long- and short-lived foci are visible. Cell contours are in green. (**E**) Schematic representation of the fate of the different categories of MutL foci. When replication errors are repaired, the focus (yellow star) is short-lived, visible in cells (red cylinders) for <4 min. When they are not repaired, the focus has a long lifetime and lasts until a new DNA replication cycle fixes the mutation on one of the two DNA molecules (23 min on average under rich growth conditions). Adapted from Reference [154].

The formation of MutL foci in *E. coli* (Figure 2A) and *B. subtilis* and PMS1 and MLH2 foci in *S. cerevisiae* depends on functional MutS or MSH2–MSH6, respectively [149,150,154] (in *E. coli* MutL foci in *mutSmutH* strain account for only 0.08% of MutL foci in *mutH* cells). The foci increase in response to the induction of mismatches in the genome in a replication-dependent manner, or when downstream MMR steps are compromised [148,149,150,151,161]. These results suggest that these foci correspond to DNA mismatch sites. The interpretation of the fate of detected mismatches varies among studies. *S. cerevisiae* and *B. subtilis* studies consider that detected MutL foci correspond to mismatches that are undergoing repair and will be successfully repaired. In *S. cerevisiae* wild-type cells, the PMS1–GFP foci have a mean lifetime of 1.5 min, whereas the MLH2–tdTomato foci are visible on average for 4 min, consistent with the idea that these foci are repair intermediates [150,151]. Indeed, newly replicated DNA in *S. cerevisiae* seems to be proficient for MMR for no longer than 10 min during S phase [162]. On the other hand, an *E. coli* study distinguishes two categories of MutL foci: long-lived and short-lived foci [154] (Figure 2D,E). Long-lived MutL foci correspond to mismatches that escape repair, persistent on DNA for 23 min on average until a new replication cycle converts them into mutations [154] (Figure 2E). Short-lived MutL foci with an average lifetime of <4 min possibly correspond to mismatches that will be repaired [154] (Figure 2E). In this sense, the result of a recent *S. cerevisiae* study investigating the localization of PMS1 and MLH2 in different MLH2 alleles showing significant MMR defect is of interest. It reports higher lifespans of PMS1–4GFP foci (eight-fold increase, mean lifespan of 25 min) and MLH2–4GFP foci in this context, and their increased frequency, possibly due to the significant increase in their lifespan. As for *E. coli* MutL foci, long-lived PMS1 and MHL2 foci could mark unrepairable mismatches converted into mutations [163]. An increased frequency of PMS1 foci correlating with the mutator phenotype of the cells, potentially due to an increased PMS1 foci lifespan, was also recently reported in an *exo1* strain overproducing CDC9 DNA ligase [164]. This study suggests that ligation of newly replicated DNA controls the timing of MMR in *S. cerevisiae* and possibly other eukaryotes.

Investigation of PMS1–4GFP and MSH6–mCherry foci in *S. cerevisiae* reported their limited co-localization, suggesting that these foci correspond to different MMR steps [150]. In contrast, an *E. coli* study found that approximately 90% of MutL foci co-localize with MutS foci [140]. The fluorescence quantification of co-localized foci enabled MutS and MutL proteins’ stoichiometry investigation. This work established that MutL focus fluorescence is always and on average 2.7-fold more intense than the fluorescence of co-localized MutS focus [140]. Furthermore, a roadblock at the d(GATC) sequences reduces the amount of MutL on the mismatch, suggesting MutL accumulation along DNA [140]. This work focused on foci formed on unrepairable mismatches in MutH endonuclease deficient cells. It is possible that the stoichiometry of MutS and MutL during efficient MMR is different from the one observed in cell that are unable to complete the repair. However, a *S. cerevisiae* study also concluded that PMS1 foci do not contain stoichiometric amounts of MSH6 by studying PMS1 foci formation in wild-type cells where the vast majority of mismatches are successfully repaired. The conclusion was based on the observation that PMS1 foci, containing on average 11 molecules per focus, are often not coincident with MSH6 foci [150]. A recent in vitro single-molecule fluorescence photobleaching study agrees with these results. MutS–MutL complexes contain up to four times more MutL than MutS [125].

Localization of MMR proteins was also investigated in mammalian cells by using immunofluorescence for endogenous proteins or live-cell imaging for fluorescently labeled proteins expressed from a plasmid [165,166,167,168,169,170,171,172,173]. However, the results remain inconsistent between the different studies. For instance, one live-cell study reported that fluorescently labeled human MSH2 forms nuclear foci that co-localize with PCNA foci [166]. Another study using the same approach did not find any foci formation [167]. Similarly, one immunostaining study reported that endogenous human MLH1 forms discrete nuclear foci [168]. Another study using the same approach found that MLH1 has a diffuse nuclear distribution [174]. Diffuse or discrete nuclear foci formation was also reported in live cells for fluorescently labeled human MLH1 proteins expressed from a plasmid [165,166,174]. The reason for these discrepancies is unclear. The expression of fluorescently tagged MMR proteins from the plasmid could affect their function and localization [165]. On the other hand, obtaining stable lines that express fluorescent MMR proteins to their native levels remains very challenging.

## 5. Exploiting MMR to Follow the Emergence of Spontaneous Mutations in Individual Cells

MMR localization in living cells is used in the MMR field to better understand the mechanism of MMR, which is done, in part, by observing the frequency of MutS and MutL foci formation in different genetic backgrounds. However, as discussed in Section 4, studies in *E. coli* show that not all MutL foci correspond to successfully repaired mismatches. Most studies of MutL foci formation do not distinguish between foci corresponding to successful repair and foci marking mismatches that will not be repaired. Observations made on the ensemble of foci are used to describe the successful MMR mechanism. However, this could be problematic, especially for accessing the kinetics of the MMR reaction.

That MutL foci also tag mismatches that are not going to be repaired, thus giving rise to mutations, was first suggested by Radman and colleagues. This idea was supported by their 2011 discovery of a linear relationship between MutL fluorescent foci frequencies in *E. coli* and mutant frequencies measured genetically over a range of several hundred times [149]. A new setup was used in a subsequent study, where cells grew continuously in steady-state conditions, and lower light, resulting in less photobleaching, allowed for the detection of fluorescent MutL foci [154]. The new study revealed the existence of two categories of MutL foci: short-lived (<4 min) and long-lived MutL foci (average lifetime: 23 min) (Figure 2D,E).

Several lines of evidence support that these long-lived MutL foci correspond to mutations. First, in cells deficient for the downstream strand incision step (*mutH* cells), hence deficient for repair, all foci are long-lived [154]. Second, in wild-type cells, where MMR repairs 99% of replication errors, while 1% gives rise to mutations, short-lived MutL foci account for 98% of foci, and ~2% only are long-lived [154]. Third, the mutation rate estimate calculated from the rate of long-lived MutL foci in MMR deficient and wild-type cells is very similar to their mutation rate previously estimated by other approaches [154,175]. Fourth, the average lifetime of long-lived MutL foci (23 min) is very similar to the average replication fork inter-arrival time (24 min) under the growth conditions tested [154]. This observation suggests that long-lived MutL foci correspond to unrepairable mismatches that persist on DNA until a new replication cycle converts them into mutations. Consistent with this, preventing new replication cycles extends the lifetime of MutL foci in MMR deficient *mutH* cells to over 200 min [149].

Because these results confirm that long-lived MutL foci correspond to mismatches that will give rise to mutations, they can be used to track the emergence of mutations in real-time in individual cells. These long-lived MutL foci point at mutations emerging in single cells before their fixation in the genome, thus independent of the effect of the mutation on cell fitness. Therefore, this approach, that exploits MMR as a tool, enables the visualization of mutations, even lethal ones, otherwise invisible in all other mutation detection approaches, because they detect mutations after their fixation. The fluorescent MutL assay can be used in combination with a microfluidic device *mother machine* [176] to allow high-throughput measurements of mutation occurrences under stable conditions (Figure 2B). The *mother machine* consists of numerous microchannels closed on one side, in which bacteria grow in a single line. The device thus allows the retention and video-microscopy monitoring of the cell immobilized at the bottom of the microchannels. Growing fluorescent MutL cells into the *mother machine* allows tracking the appearance of spontaneous mutations in thousands of single-cells in parallel over more than 100 cell divisions (Figure 2C,D). In other words, the experiment (that follows in living cells, cell by cell, over long periods of time, mutation occurrences in *E. coli*) somehow fulfills Luria and Delbrück’s dream 75 years after their seminal experiment demonstrating that spontaneous mutations occur in the absence of selection, rather than as a response to selection.

Studying mutations in single cells and in real time allows us to investigate aspects of the mutation process that were previously out of reach. For example, through this approach, we can directly access the dynamics of mutations, i.e., ask whether the occurrence of spontaneous mutation, as assumed by Luria and Delbrück, is a Poisson process. In other words, do spontaneous mutations occur at a constant and homogeneous rate in the population? If not, what distinguishes subpopulations with different mutation rates? With this tool, it is now possible to relate a creation of mutation to single-cell gene expression. Fluorescent MutL assay therefore allows quantitative studies of mutation rate variability in isogenic populations, providing a means to access its sources and consequences, and providing new insights to the mutagenesis field. The process of mutation can now be added to a growing number of cellular processes for which it has recently become possible to study fluctuations among single cells.

The new mutation-detection tool based on fluorescent MutL was used in three studies so far. Two processes that control spontaneous mutation arising from replication errors are error production and error repair. Investigating MutL foci in MMR-deficient cells, inactivated at a step following MutL binding to MutS, allows us to access the first of these processes, while the MMR efficiency investigation requires us to use a wild-type strain with a functional MMR system. The error production was investigated first, in a study that investigated the appearance of MutL foci in MMR-deficient *mutH* cells growing in the *mother machine*. This work showed that the time intervals between two successive MutL foci are exponentially distributed, as expected for the Poisson process. Furthermore, a small fraction of cells exhibiting a reduced growth rate and/or abnormally large cell size, indicating some level of endogenous stress did not exhibit higher error production (i.e., MutL foci) than the rest of the cells in a normally growing population. These findings ruled out strong and moderate variations between cells in the rate of error production.

In a separate work, Ivan Matic and colleagues used the fluorescent MutL assay to investigate error production in single cells [152]. Instead of growing cells in a microfluidic chip and following them by video-microscopy, they took snapshot images of cells plated on agarose pads and expressing fluorescent MutL and different stress reporters. This study aimed to investigate the impact of cell-to-cell variability in stress responses induction and spontaneous translation errors on replication error production. They found that cells with the highest induction of SOS, the highest heat-shock induction, or the highest level of translation errors have more replication errors compared to cells with the weakest stress signal or the lowest level of translation errors. Their results indicate that replication errors arise more frequently in subpopulations with unique phenotypic properties, suffering from endogenous stresses. The discrepancy between the results of the two studies could be due to the fact that the first study only examined stress levels impacting cell growth or morphology. The second study may have investigated lower stress levels not affecting cell growth and size. Another difference is that the first study compared the rate of MutL foci per replication fork for different subpopulations. In contrast, in the Matic laboratory study, the average number of MutL foci was normalized to cell size, not replication cycle. Finally, the group of Stephan Uphoff used a fluorescent MutL assay and microfluidics to follow MutL foci in wild-type cells in response to mismatch-inducing DNA alkylation treatment [153]. In this study, fluorescent MutL foci and Ada promoter expression were monitored simultaneously in single cells to relate mutagenesis to the ability of the individual cell to repair DNA alkylation damage. The study showed that the rate of MutL foci upon treatment was negatively correlated with the level of Ada and positively correlated with the time of Ada induction in single cells. Random variation in the activation of Ada response between cells in response to the mismatch-inducing treatment resulted in heterogeneity in the rate of MutL foci between cells in an isogenic population.

Further studies will examine whether the mutation rate is heterogeneous in isogenic wild-type populations not stressed by external factors and whether heterogeneity in the mutation rate could be evolutionary beneficial to populations.

## 6. Conclusions

The study of MMR reconstituted in vitro has significantly advanced our understanding of the MMR mechanism. Visualization of MMR complexes in live cells completes this comprehension by interrogating the MMR mechanism in the complex cellular environment. Unlike in vitro reconstituted MMR systems, in living cells, MMR works in highly heterogeneous environments in the context of chromatin structure and dynamics. It is coupled to DNA replication, interacts with other DNA repair systems, and has a (replication) defined timing for repair. Most efforts to date have focused on the initial steps of MMR. Future in vivo MMR imaging could one day enable observation of the entire reaction: visualization of the different intermediates, characterization of their dynamics, and determination of the stoichiometry of proteins in the repair intermediates. In particular, in the step following mismatch recognition, MutL associates with MutH and UvrD, and the stoichiometry of these interactions is entirely unknown. Classical epifluorescence has limited potential in this regard. Due to its limited spatial resolution, it cannot discriminate true complex formation from simple co-localization. Furthermore, protein accumulation can be observed with this approach but not the presence of a single-molecule transiently dwelling at some location in the cell. Single-molecule FRET in living cells could make this possible.

In addition to mechanistic aspects, future imaging of MMR complexes may challenge the universality of the mutation detection method based on MutL visualization. If applicable to mammalian cells, it may be worthwhile to employ it for monitoring mutations in small transparent animals. For instance, animal lines expressing MutL–homologue–GFP could be constructed and allow monitoring mutagenesis in different cell types during normal development or pathogenic processes. In repair proficient cells, mutations occur as a result of repair failure. Detecting, in real time, the emergence of spontaneous mutations in living cells may provide valuable insight into why some errors are not repaired and how the efficiency of MMR varies between identical cells. Furthermore, it allows us to interrogate the causes of the heterogeneity in MMR efficiency and their potential consequences for the adaptability of populations.
